# Cumulative adversity, mindfulness, and mental health in first-time mothers experiencing low income

**DOI:** 10.1016/j.jadr.2023.100621

**Published:** 2023-06-28

**Authors:** Luciano Garofalo, Cathryn Booth-LaForce, Paula Nurius, Stephanie Thompson, Becca Calhoun, Lisa Shimomaeda, Liliana Lengua

**Affiliations:** a University of Washington, Department of Child, Family, and Population Health Nursing, United States; b University of Washington, School of Social Work, United States; c University of Washington, Center for Child and Family Wellbeing, United States; d University of Washington, Department of Psychology, United States

**Keywords:** Childhood adversity, Prenatal mental health, Poverty, Mindfulness

## Abstract

**Background:**

Socioeconomic adversity can negatively impact the mental and physical health of mothers and their offspring. This paper extends understanding of the interrelationship of these factors during the perinatal time frame, specifically to: 1.) evaluate the impact of adverse childhood events (ACES), negative life events (NLEs), and socioeconomic factors on the mental health of low-income expecting mothers; 2) evaluate for differences by race/ethnicity in adversity predicting perinatal mental health; 3) examine how mindfulness interacts with socioeconomic adversity in predicting prenatal mental health.

**Methods:**

202 predominately non-white primiparous females with household income at or below 200% of the federal poverty level participated. Measures included ACEs, NLEs, and several descriptors of socioeconomic status. Resilience and mindfulness were measured as possible protective factors. In the sequence of socioeconomic factors, ACEs, and NLEs, hierarchical multiple regression tested these measures as predictors of prenatal depression and anxiety, also evaluating for interaction effects.

**Results:**

Controlling for socioeconomic factors, higher ACEs and NLEs independently predicted worse prenatal mental health. Cumulative socioeconomic risk interacted with ACEs to predict higher prenatal anxiety and more accurately predict prenatal depression scores. Higher mindfulness predicted lower anxiety and depression regardless of adversity, but was also significantly negatively correlated with ACEs.

**Limitations:**

Generalizability of results are limited by the sample size and specific geographic region. Conclusion: Socioeconomic and psychosocial adversity cumulatively impact prenatal mental health. Mindfulness may be a valuable target for resilience-enhancing interventions prepartum but is not a solution to the clear detriments that social and economic marginalization have on mental health.

## 1. Introduction

Perinatal anxiety and depression diagnoses increased between 2006 and 2015 from 18.4 to 40.4 per 1000 deliveries in the US (McKee et al., 2020) and are associated with negative outcomes for both mothers and offspring (Rogers et al., 2020). Pregnancy and the postpartum period are inherently stress-inducing, the differential impact of which is determined by one’s preexisting stress and vulnerability to new stressors. Evidence from high-income and low- to middle-income countries consistently implicate socioeconomic hardship and social/relational hardship (e.g. domestic violence, migrant status) as strong risk factors for perinatal mental disorders (Howard et al., 2014). Such hardship can have detrimental and cumulative effects on several domains, including attachment/relationships, education, employment, and physical health (Nurius et al., 2015). Adverse childhood experiences (ACEs) include various forms of abuse, neglect, or household instability experienced before age 18, and their associated health consequences have been studied since 1998 when the landmark ACE study was conducted (Felitti et al., 1998). A higher burden of ACEs is predictive of adverse experiences in adulthood, including worse mental and physical health and greater SE hardship (Gilbert et al., 2015; Nurius et al., 2015; Labrenz et al., 2020; Youssef et al., 2017; Slack et al., 2017).

Socioeconomic adversity and trauma can be especially burdensome in parenthood (Katz et al., 2018; Borja et al., 2019). Consequently, parental stress and traumatic/adverse experiences can negatively impact the highly plastic neural networks in a developing child, such as through conditioning of the autonomic nervous system (Thompson et al., 2019) and reduced volume of the frontal and temporal lobes (Teicher et al., 2016). Higher susceptibility to stress is conferred through epigenetic and developmental changes inherited by subsequent offspring (Bale, 2014; Chan et al., 2018). These changes are known to be maladaptive in the long term, affecting behavior, socialization, and mental and physical health (Rotheram-Fuller et al., 2018; Zvara et al., 2017; Thomas JC et al., 2018; Gray et al., 2017). The pathway from ACEs to future adverse experiences thus is cyclical and expansive within family units.

A growing body of evidence demonstrates how xenophobia, structural racism, and historical trauma contribute to a higher burden of ACEs on people of color in the US, impacting mental and physical health outcomes (Labrenz et al., 2020; Kim et al., 2020; Slack et al., 2017; Youssef et al., 2017; McClendon et al., 2021; Polanco-Roman et al., 2021). Systemic inequalities in multiple sectors such as housing, education, employment, and financing can keep families of color stuck in a cycle of poverty, with associated detriments on health and wellbeing. The experience of discrimination can result in stress and trauma that compound these effects on one’s health, and have negative consequences on health behavior and healthcare utilization due to fractured trust in institutions (McClendon et al., 2021; Powell et al., 2019).

While both poverty and ACEs are associated with worse health status in adulthood, a study using Behavioral Risk Factor Surveillance System (BRFSS) data in Wisconsin showed the effects of race fell away when controlling for these factors in its model, despite higher total ACEs and greater poverty reported by Black respondents compared to White respondents (Slack et al., 2017). A similar effect was observed in another study on health-related quality of life among fifth-graders (Wallander et al., 2012). However, using national BRFSS data from 2010, Labrenz et al. recently found when modeling the interaction of race/ethnicity and ACEs on days inhibited by mental health problems, this interaction fully attenuated the independent effect of ACEs on mental health (Labrenz et al., 2020). For certain non-White populations in the United States, higher ACE scores may have a more pronounced detrimental effect on mental health. For example, Black pre- and postpartum mothers in Hennepin County, Minnesota, were much more likely to report to have experienced racism, witnessed violence, and lived in unsafe neighborhoods compared to White mothers (Kim et al., 2020). While higher ACEs were associated with worse mental health in all mothers in this sample, Black mothers and mothers of color had significantly more instability in food, housing, and transportation than White mothers.

These results illustrate the well-established yet still overlooked fact that race itself is not a health risk factor; race is a social construct that is contextualized by the life course. It is important to more granularly understand elements of the lived experience that may interact with elements of identity to determine their differential impact on one’s health. Systemic racism and xenophobia conflate the socioeconomic adversity that people of color and immigrants often experience, which may be compounded by experiences of discrimination to the detriment of one’s health. Conversely, adversity may beget adaptive traits that protect one who has weathered much from certain negative health consequences. To effectively address mental health problems in pregnancy, it is critical to understand the parts that make up the whole by assessing and supporting personal and interpersonal strengths in addition to psychopathology (Tse et al., 2016).

Resilience is the ability to withstand, adapt to, or recover from challenges, and is also a theoretically heritable trait. Resilience is a known protective factor from negative health effects of childhood adversity, which is contextualized by one’s unique lived experience (Bethell et al., 2016; Ellis & Dietz, 2017). Furthermore, adversity experienced by parents may sometimes beget traits of resiliency in their offspring and buffer the negative impacts of other adverse experiences (Taylor et al., 2020). Howell et al. recently studied a diverse sample of low-income expecting mothers and found that resilience traits may buffer the impact of ACEs on mental health, likely benefitting both mother and offspring (Howell et al., 2020). Their study differentiated between individual, relational, and contextual resilience using the validated *Resilience centre Research Adult Resilience Measure* and found that relational resilience was the only statistically significant protective mediator between ACEs and prenatal depression.

Resilience-promoting traits such as mindfulness may be important targets in strengths-based approaches to mental health care, especially during pregnancy. Mindfulness is characterized by an adaptive mental state where present experiences are met with an attitude of non-judgment. Mindfulness training can be beneficial for perinatal mental health, though most of the existing evidence is limited to small randomized-controlled trials (Dimidjian et al., 2016; Duncan et al., 2017). Most follow-up in related trials is limited to the early postpartum period, but there is preliminary evidence for mental health benefits of mindfulness training in pregnancy extending beyond 12 months postpartum (Shi and MacBeth, 2017; Felder et al., 2018; Sbrilli et al., 2020). Higher mindfulness in mothers may also positively influence attachment and social-emotional development in offspring (Pickard et al., 2017). However, very little research has investigated the role of mindfulness in mental health for mothers experiencing socioeconomic adversity.

The present study addresses three gaps in the literature. First, given the highly intertwined nature of socioeconomic factors that can negatively impact perinatal mental health, there is a need to better understand the relations among these factors and the magnitude of their effects. Second, past studies evaluating ACEs and their impact on perinatal mental health have involved samples of mothers who are mostly white and middle class. The present study includes a robust set of socioeconomic assessments in a cohort of mothers living in the context of low-income who were predominantly people of color (Thompson et al., 2022; Lengua et al., 2023). Third, mindfulness is an important aspect of perinatal mental wellbeing but is under-studied in pregnant people, people with low-income, and people of color. We aimed to: 1) evaluate the impact of ACES, negative life events, and several socioeconomic factors on the mental health of low-income expecting mothers; 2) to evaluate for differences by race/ethnicity in adversity predicting perinatal mental health; and 3) examine how mindfulness interacts with socioeconomic adversity in predicting prenatal mental health.

## 2. Methods

This study was conducted with pre-treatment data from the NEW Moms Connect (Nurturing Emotional Well-being) trial, an investigation of several interventions addressing perinatal wellbeing and parenting on the neurobiological development of low-income mothers’ offspring (Thompson et al., 2022; Lengua et al., 2023). The protocol was approved by the Institutional Review Board at the University of Washington and informed, signed consent was obtained from all mothers prior to participating in study procedures.

### 2.1. Participants

Participants were recruited from local hospitals, clinics, and community organizations from February 2017 to June 2019. Medical providers and support staff were trained to promote the study to potentially eligible patients, who then expressed interest via phone or email. To be eligible, participants had to be primiparous females, aged 18 or over, with a household income less than 200% of the US Federal Poverty Level (operationalized as less than $45,000 annual income for a family of three). Participants were required to be fluent in English to the extent that they could participate in assessments and classes conducted in English. Exclusion criteria included mothers who: 1) were beyond 33 weeks’ gestation; 2) self-reported addiction to alcohol or other substances; 3) self-reported hallucinations or an altered sense of reality; 4) had previously given birth; 5) were under age 18; 6) were pregnant with multiples. The present study was a secondary data analysis of data collected as part of the study evaluating the effects of perinatal mindfulness and parenting interventions on the mothers’ and their infants’ mental health (Lengua et al., 2023). The exclusion criteria were relevant to women’s ability to participate in the interventions and assessment of intervention effects. The sample size for the parent study was determined to have sufficient power to detect small intervention effects.

### 2.2. Measures

**Psychosocial stress** was captured through the *Adverse Childhood Experiences Questionnaire* and number of current *Negative Life Events* (NLE). The ACEs Questionnaire produces a continuous score based on adverse events that occurred in the first 18 years of life. It includes ten dichotomous *yes/no* questions such as “Did a household member go to prison?” and “Were your parents ever separated or divorced?”; as well as four questions regarding the frequency of events such as “How often, if ever, did you see or hear someone being beaten up, stabbed, or shot in real life?” (*Many times, A few times, Once, Never, Refused;* Felitti et al., 1998). Participants also reported on their experience of major life events (NLE) such as moving, major conflicts, and legal problems using the 18-item General Life Events Schedule (Sandler et al., 1986), which includes moderately and highly stressful life events, such as moving, losing a job, or death of a family member or friend. Respondents indicated whether each of the events occurred in the past year, and scores were the number of events endorsed.

**Ethnicity and race** were categorized as factor variables according to their Centers for Disease Control and Prevention concept codes (*Ethnicity* = *Hispanic/Latino or Not Hispanic/Latino; Race* = *African/African American/Black, Asian, Native Hawaiian/Pacific Islander, Native American Indian/Alaskan Native/Indigenous, Caucasian/White, Other*). Participants who chose more than one race were recoded as “Other”.

Numerous **socioeconomic factors** were collected to better characterize potential mechanisms of economic or societal adversity. **Financial Insecurity** was assessed using the average of seven items representing the degree to which participants had enough money to afford living essentials (e.g., home, food, medical care, etc.; Conger et al., 1994) reported on a 5-point Likert scale (5 = *strongly disagree* to 1 = *strongly agree; M* = 3.43, *SD* = 0.83, α = 0.87). **Highest level of education completed** was a categorical variable (*1* = *11th grade or less; 2* = *High School or GED; 3* = *Two years of college or technical school; 4* = *College degree,* e.g. *BA, BS; 5* = *Masters degree or higher*), with *Less than high school education* (*Yes/No*) being treated as a separate dichotomous risk factor. Similarly, **housing status** was coded as a categorical variable (*1* = *Own home; 2* = *Rent; 3* = *Live with extended family; 4* = *Temporary housing; 5* = *Homeless*), while *Unstable Housing* (*Yes/No*) was a separate dichotomous risk factor, defined by living with extended family, living in temporary housing, or homelessness. **Housing density** was represented with a proportion score based on number of people per room. Finally, two dichotomous variables were included indicating whether or not the mother was a **single parent** or an **adolescent** (18 year or younger).

A **cumulative risk score** indicating the accumulation of stress and adversity in the current context was derived from a sum of the six risk variables: adolescent parent status, single parent status, maternal education, household density, housing insecurity, and negative life events. Dichotomous factors (adolescent and single parent status, low maternal education, housing insecurity) were scored as 0 = *not present*, 1 = *present*, whereas continuous factors (negative life events, household density) were converted into a proportion score (i.e., proportion of total possible) ranging from 0 to 1.

**Resilience** was measured according to the Brief Resilience Scale (BRS; Smith et al., 2008), a 6-item self-assessment of one’s ability to recover from stressful experiences. The BRS uses a 5-point scale ranging from 1 (*strongly disagree*) to 5 (*strongly agree*) indicating the extent to which participants agree or disagree with statements such as *“I tend to bounce back quickly after hard times.”* The measure demonstrates adequate internal consistency and validity, and is distinctive from constructs of depression, anxiety, and stress (Kyriazos et al., 2018). The alpha for the current study was 0.84.

**Mindfulness** was measured with the Five Facet Mindfulness Questionnaire-Short Form (FFMQ; Baer et al., 2006). 24-items were completed on a 5-point Likert-type scale (1 = *never or very rarely true*, 5 = *very often or always true*), capturing the extent to which individuals considered themselves to practice observing, describing, and acting with awareness, non-judgmentally and non-reactively. The FFMQ is the most widely utilized measure of mindfulness (de Bruin et al., 2012) and the short-form has been found to be a reliable and valid measure (Bohlmeijer et al., 2011). The alpha for the current study was 0.89.

**Mental health measures** included a *Questionnaire for General Anxiety Disorder* (GAD-7; Spitzer et al., 2006) and the *Center of Epidemiologic Studies measure for depression* (CES-D; Radloff, 1977). The GAD-7 assesses the frequency with which one experienced anxiety symptoms such as “Feeling nervous, anxious, or on-edge” in the past two weeks (0 – *Not at all; 1 - Several days; 2* – *More than half the days; 3* – *Nearly every day*). Similarly, the CES-D poses statements descriptive of depression symptoms such as “I felt lonely” and assesses the frequency with which one experienced them in the past week (*Less than 1 day; 1*–*2 days; 3*–*4 days; 5*–*7 days*). Both were collected after study enrollment in prepartum and again at 2–4 months postpartum. The alphas for the GAD-7 and CES-D for the current study were 0.85 and 0.87, respectively.

### 2.3. Analysis

Data were analyzed using SPSS and R Studio (RStudio Team 2020). Descriptive statistics were produced to examine sample characteristics. To answer the first research question regarding the impact of childhood and adulthood adversity on prenatal mental health, we evaluated zero-order correlations between all measures. We then performed a hierarchical multiple regression, sequentially regressing socioeconomic factors and maternal stress factors onto the outcomes of depression and anxiety in the prenatal period. The order of factor entry to the multiple regression was determined through a life course perspective, following the observation that adversity in adulthood often stems from and is predicted by early life hardship in the socioeconomic family environment (Borja et al., 2019). Model residuals were plotted against the predicted fitted values. Because some heteroscedasticity was observed, model coefficients were calculated with robust standard errors and 95% confidence intervals. The cumulative risk score and ACE score were then regressed onto depression and anxiety along with an interaction term (cumulative risk * ACEs) to test for effect modification of these adversities on mental health.

To answer the second research question regarding differences by race/ethnicity in adversity predicting prenatal mental health, we evaluated for differences between racial/ethnic groups in perinatal stress, mental health, and SE factors using a factorial analysis of variance (ANOVA). Due to small numbers of participants identifying as Native Hawaiian/Pacific Islander and Native American Indian/Alaskan Native/Indigenous, these race categories were also recoded into “Other” for the regression models. We used Tukey post-hoc tests to determine pairwise differences between non-Hispanic white mothers and other racial/ethnic groups in ACEs, NLEs, and mental health outcomes.

For the third research question regarding how mindfulness contributes to prenatal mental health in the context of adversity, mindfulness, cumulative risk, and ACEs were each regressed onto prenatal depression and anxiety. Controlling for cumulative risk, mindfulness and ACEs were multiplied together to create the interaction term. depression and anxiety were regressed on the predictors and interaction term to test for effect modification. Beta-coefficients and p-values are presented for all models. All effect sizes are interpreted herein according to Cohen’s criteria (Cohen, 1988).

## 3. Results

Baseline characteristics of all participants are summarized in Table 1. Approximately one-third of the sample identified as white and one-third identified as black. 20% of mothers identified as Hispanic/Latinx. 39% of mothers were single, 22% reported unstable housing, and all but 6% of mothers had completed high school. Compared to white mothers, a significantly greater proportion of black mothers were single parents (49% vs 29%; *p* < .01) and had greater housing density. Significantly fewer Hispanic/Latinx mothers were single (15% vs 38%; *p* < .05) and had more stable housing than non-Hispanic/Latinx mothers. There were no other statistically significant differences in socioeconomic factors between racial/ethnic groupings.

Total ACE scores were significantly lower for black mothers (2.4 vs. 3.3; *p* < .05) and significantly higher for mothers identifying as American Indian/Alaskan Native/Indigenous (6.4 vs. 3.3; *p* < .05) compared to white mothers. Tukey post hoc test for pairwise comparisons of ACE scores between racial groupings confirmed significantly lower total ACE scores for black mothers compared to both AIAN mothers and mothers identifying as Other racial groups. Black mothers also had higher resilience scale scores (21.7 vs. 20.6; *p* < .05) compared to white mothers.

We analyzed zero-order correlations among all study variables (see Supplement Table 1). There was a significant negative association between reported financial security and prenatal depression scores (*r* = −0.278; *p* < .01) and anxiety scores (*r* = −0.199; *p* < .01). ACE scores and number of negative life events had moderate positive associations with prenatal depression (ACE: *r* = 0.341; NLE: 0.413; *p* < .01) and anxiety symptoms (ACE: 0.359; NLE: 0.393; *p* < .01). Unstable housing was associated with higher prenatal depression scores (*r* = 0.155, 0.164, respectively; *p* < .05). Higher reported resilience and social satisfaction were strongly negatively associated with all perinatal depression and anxiety scores.

Results of the full hierarchical multiple regression are depicted in Table 2. All the predictor sets produced a significant additive change in *R*^*2*^, representing cumulative contributions to the outcomes of depression and anxiety scores. Controlling for race and other socioeconomic factors, lower financial security predicted worse prenatal depression and anxiety symptoms. However, the effect of financial security became nonsignificant with the introduction of ACEs and NLEs into the model, respectively. When introduced to the regression, higher ACE scores significantly predicted worse prenatal depression and anxiety symptoms (β = 1.15, 0.60, respectively; *p <* .001). Negative life events also strongly predicted worse prenatal depression and anxiety symptoms (β = 0.95, 0.41, respectively; *p <* .001). ACEs remained significant for worse prenatal mental health with introduction of NLEs into the model indicating each was accounting for unique variance in outcomes.

Having less than high school education had a surprisingly large magnitude of effect on higher prenatal depression scores, which was maintained despite the addition of ACEs and NLEs. Less than high school education was also predictive of higher anxiety scores when controlling for other socioeconomic factors, but this effect became nonsignificant with introduction of ACEs and NLEs.

**Fig. 1.**
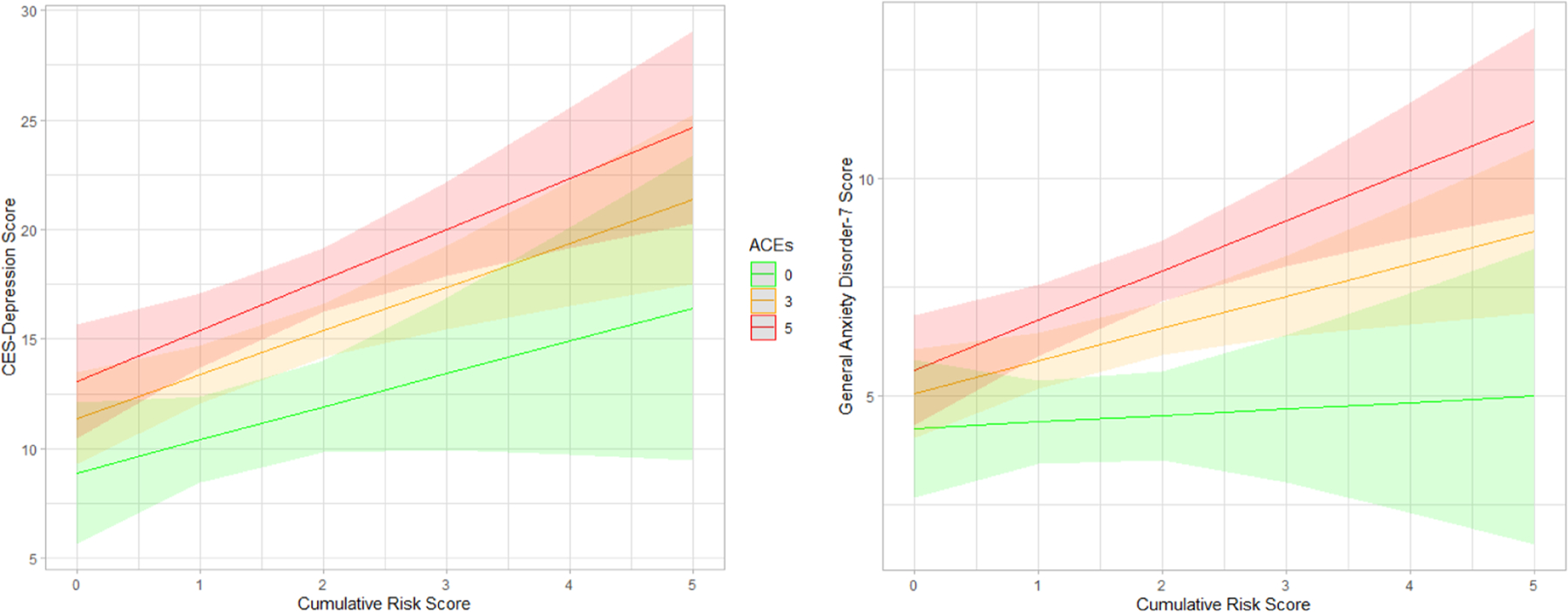
Predicted depression and anxiety scores by cumulative socioeconomic risk and ACEs.

In model 2, cumulative socioeconomic risk and ACEs both strongly predicted worse prenatal depression and anxiety symptoms (Table 3). When testing for effect modification, ACEs and cumulative risk interacted to predict increasingly worse anxiety (Table 3; Fig. 1). This effect was not observed for prenatal depression symptoms, but measuring cumulative risk and ACEs together seemed to generate a more robust estimate (Fig. 1).

**Fig. 2.**
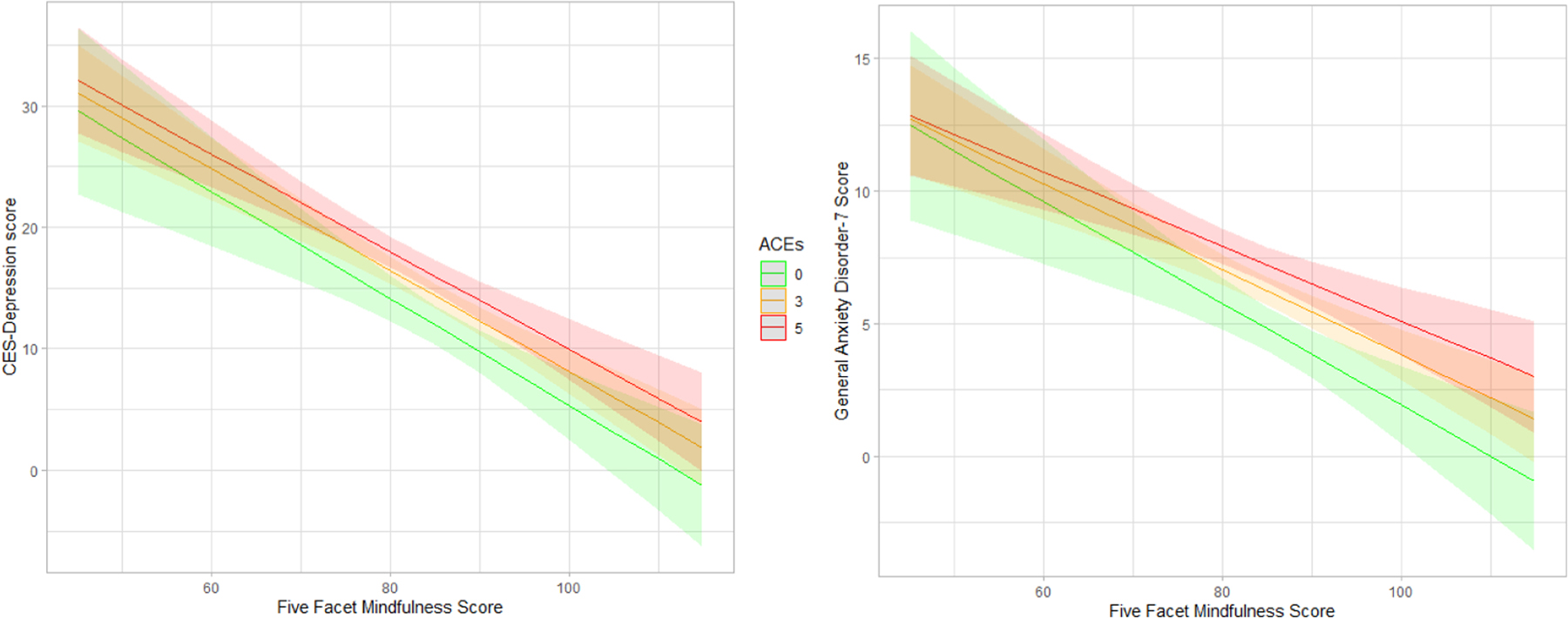
Predicted depression and anxiety scores by mindfulness and ACEs.

Model 3 included mothers’ mindfulness and ACEs as cofactors regressed on prenatal anxiety and depression scores. Mindfulness independently and strongly predicted less depression and anxiety regardless of ACE score (Table 4). No interaction between the two variables was observed for prenatal depression or anxiety symptoms, although mothers’ mindfulness appeared slightly less effective at attenuating prenatal anxiety when ACE score was 5 or greater (Fig. 2).

## 4. Discussion

We evaluated multiple layers of adversity on perinatal mental health with a sample of first-time mothers who all had low-income and were primarily women of color. As expected, ACEs and negative life events both strongly predicted prenatal depression and anxiety symptoms in addition to socioeconomic factors. The finding of lower reported ACE scores for black mothers differs from several prior studies with US national sampling where higher ACE scores were found in black racial groupings (Labrenz et al., 2020; Merrick et al., 2018). These data offer little context for this observed difference, other than black-identifying mothers having higher reported resilience than white mothers. Roughly ¼ of black mothers in our sample were born outside the US, which is notable because several prior studies have demonstrated that first-generation US immigrants have reported lower mean ACEs than second-generation and Native-born Americans (Caballero et al., 2017; Vaughn et al., 2017). The first-generation black mothers in this sample reported lower mean ACE scores than US-born black mothers, but the difference was not statistically significant (1.71 vs 2.38; *p* = .27).

Having less than a high school education also predicted prenatal depression symptoms, independent of ACEs and negative life events. While the directionality of this relationship cannot be determined due to this study’s cross-sectional design, this finding amplifies the call for more attention and resources for mental health treatment in adolescents. Addressing mental health, particularly through resilience-building earlier in the lifespan, would have long-term individual and intergenerational benefit, given what is understood about the impact of parental mental wellbeing on child neurobiological development.

Besides education level, no other socioeconomic factors predicted better or worse prenatal mental health when adjusting for traumatic experiences. However, the continuous sequential improvement to our model with each additive step of predictors indicates that our understanding of prenatal depression and anxiety symptoms would be incomplete without them. Furthermore, cumulative socioeconomic risk, representing the current context of risk, interacted with ACEs, which assess childhood experiences of risk, to predict greater prenatal anxiety symptoms, and the two measures modeled together may generate a more robust estimate of prenatal depression symptoms. These findings provide novel insight to the compounding effect that childhood adversity and current socioeconomic adversity have on prenatal mental health in this under-researched population. An adequate health risk assessment in the prenatal period must assess socioeconomic adversity, traumatic experience, and resilience factors through a life-course perspective.

We also observed that mindfulness was moderately protective for prenatal mental health regardless of cumulative risk or ACE burden. A recent systematic review summarized the evidence for clinical studies targeting internal resilience factors (e.g. self-esteem, mindfulness, active coping) for antenatal depressive symptoms, which primarily included cognitive behavioral therapy (CBT) based and mindfulness-based interventions (MBI; Walker et al., 2022). Although bias was a concern for the included studies, MBIs were generally efficacious in reducing depressive symptoms in several different ethnic populations, as well as improving secondary study outcomes of mindfulness, acceptance, coping, and self-esteem (Walker et al., 2022). A separate systematic review and meta-analysis evaluated specifically the effect of MBIs on perinatal mental health for those with and without current mental health issues (Yan et al., 2022). Despite the highly variable quality of evidence, this study also found MBIs to be useful for those with current depression and/or anxiety after pooled results analysis (Yan et al., 2022).

### 4.1. Strengths/limitations

Strengths of this study include the racial/ethnic diversity of the sample and the robust socioeconomic status measures that were collected. Although the sample size is a strength in the context of the NEW Moms Connect prospective cohort investigations, published elsewhere, it is a limitation in the context of this study due to its cross-sectional design. We did not have sufficient power to test the interactions of race with other factors, such as to reproduce the analysis performed by Labrenz et al. (2020). While we combined ACEs, NLEs, and socioeconomic measures to investigate differences in adversity potentially resulting from structural racism, these are only partial and proxy measures of a complex lived experience. There are also notable regional differences in culture and institutions in the US that can present different opportunities and challenges for people of color. An additional limitation of this study is the reliance on participants’ reports on all measures, increasing the likelihood that shared method variance might account for observed associations.

### 4.2. Clinical implications

Exposure-based therapies like CBT and interpersonal psychotherapy have been criticized as being less-than-ideal in pregnancy because of their observed low adherence and high attrition in the literature, as well as the neuro-endocrine effect of confrontation in anxiety states possibly driving negative birth outcomes (Walker et al., 2022; Arch et al., 2012; Burger et al., 2020). For expecting mothers with mild-to-moderate depression and/or anxiety symptoms, resilience-building therapies such as mindfulness training may be favored over exposure-based therapies like CBT. However, while resilience-enhancement is valuable, socioeconomic adversity and trauma are the most impactful factors driving psychosocial distress for expecting mothers.

To address the root cause of health disparities in low-income communities and people of color in the US, systemic change via policy and economic resources remain the most relevant tools. Regarding clinical intervention, resilience-building through mindfulness may be beneficial for expecting mothers facing multiple levels of adversity. Unfortunately, most mindfulness-based interventions in the West are designed and taught from a White-centric perspective and tested in more privileged populations (Proulx et al., 2018; Watson-Singleton et al., 2019), thus remaining naïve to the differential experience of people of color. More study is needed to develop culturally acceptable and effective mindfulness-building interventions for specific populations.

## 5. Conclusion

In this sample of primiparous females with low income, adversity in the form of ACEs and NLEs were both predictors of higher prenatal depression and anxiety scores, independent of several socioeconomic factors. ACEs interacted with cumulative socioeconomic risk to predict higher prenatal anxiety and more accurately predict prenatal depression scores. Having less than high school education was the only socioeconomic predictor of higher prenatal depression scores when controlling for other measures of adversity. Mindfulness may be a valuable target for resilience-enhancing interventions prepartum but is not a solution to the clear impact that social and economic marginalization have on perinatal mental health.

## Supplementary Material

Supplementary Material

## Figures and Tables

**Table 1:** Study participant characteristics and comparisons across racial/ethnic grouping.

	n (%)	Mean ACE	Mean NLE	Mean Financial security	Single parent (%)	Less than HS education (%)	Adolescent (%)	Unstable Housing (%)	Mean Household Density	Mean BRS	Mean SSQ	Mean Self- reported Mindfulness
**White**	59 (29.1)	3.3	6.5	3.3	17 (29)	5 (9)	5 (8)	12 (20)	0.22	20.6	31.7	83.8
**Asian**	27 (13.3)	3.8	5.5	3.1	10 (37)	0 (0)	2 (7)	4 (16)	0.28	19.9	29.8	79
**Black**	73 (36.0)	**2.3** *	6.1	3.5	**36 (49)** *	4 (6)	4 (5)	18 (25)	**.29** *	**21.8** *	32.1	85.9
**Other**	43 (21.2)	4.5	6.6	3.4	13 (30)	3 (7)	2 (5)	10 (23)	0.26	20.4	31.9	83
**Hispanic (vs. non-Hispanic**	40 (19.7)	**4.3** *	6.5	**3.5**	**6 (15)** *	3 (8)	1 (3)	5 (13)	0.23	20.5	31.8	81.5
**TOTAL / MEAN**	203	3.3	6.3	3.4	76 (39)	12 (6)	13 (6)	44 (22)	0.26	20.9	31.7	84

HS = high school; ACE = adverse childhood events; NLE = Negative Life Event; BRS = Brief Resilience Scale; SSQ = Social Satisfaction Questionnaire.

* *p* ≤ 0.05.

**Table 2:** Model 1: Hierarchical regression demonstrating unique and additive effects of risk factors as predictors of prenatal depression and anxiety.

	Depression			Anxiety		
	Step 1	Step 2	Step 3	Step 1	Step 2	Step 3
F	**3.23**	**5.28**	**6.52**	**2.40**	**4.63**	**5.43**
R^2^	**0.10**	**0.20**	**0.26**	**0.07**	**0.17**	**0.22**
Age	0.04	0.04	0.08	0.04	0.04	0.05
White	ref	ref	ref	ref	Ref	ref
Black	0.87	1.80	2.18	−0.86	−0.38	−0.22
Asian	2.07	1.70	3.00	−1.01	−1.20	−0.64
Other race	1.33	0.53	0.90	−1.48	**1.90** *	−1.74
Hispanic/Latinx	−1.01	−1.65	−1.57	0.90	0.57	0.60
<HS education	**7.05** *	**5.96** *	**5.79** *	**2.64** *	2.08	2.00
Single parent	1.36	1.30	0.71	0.69	0.65	0.40
Financial security	**2.82** *	**2.23** *	−1.27	**0.97** *	−0.66	−0.25
Housing density	1.84	1.29	0.47	1.36	1.08	0.72
Unstable housing	1.12	0.83	0.48	0.96	0.81	0.66
ACE score		**1.15** **	**0.84** **		**0.60** **	**0.46** **
NLE			**0.95** **			**0.41** **

Terms in all rows below R^2^ are regression (β) coefficients. HS = high school; ACE = adverse childhood events; NLE = Negative Life Events.

* *p* < .05;

** ***p* < .001**.

**Table 3:** Model 2: Cumulative socioeconomic risk and ACEs predicting prenatal depression and anxiety.

Terms	Depression (β)		Anxiety (β)	
	Step 1	Step 2	Step 1	Step 2
Cumulative risk	***2.07*** **	1.51	**0.84** *	0.15
ACEs	***1.08*** **	**0.83** *	***0.57*** **	0.27
Cumulative risk * ACEs	-	0.16	-	0.20

* p *<* 0.05;

** *p <* .001.

**Table 4:** Model 3: Mindfulness and ACEs predicting prenatal depression and anxiety.

Terms	Depression (β)		Anxiety (β)	
	Step 1	Step 2	Step 1	Step 2
Mindfulness	−0.41**	−0.44**	−0.16**	−0.19**
ACEs	***0.80*** **	0.16	***0.47*** **	−0.38
Mindfulness * ACEs	-	0.01	-	0.01

* p *<* 0.05;

** *p <* .001.
