# Supplementary material for: Cumulative adversity, mindfulness, and mental health in first-time mothers experiencing low income
Source: J Affect Disord Rep. Author manuscript; Available in PMC 2025 Aug 15. (PMC12352410; doi:10.1016/j.jadr.2023.100621)
Supplement: Supplementary Material [file NIHMS2046189-supplement-Supplementary_Material.docx]

| **Supplement 1: Zero-order Correlations of all study variables** | | | | | | | | | | | | | | | | | |
| --- | --- | --- | --- | --- | --- | --- | --- | --- | --- | --- | --- | --- | --- | --- | --- | --- | --- |
|  | | | | | | | | | | | | | | | | | |
|  | | | | | | | | | | | | | | | | | |
|  | | financial security | Age | Adolescent | Single Parent Status | Negative Life Events: number | ACES total | Highest level of education completed: | Number of people in household | Household Density (ppl/rooms) proportion score | Unstable Housing Status | Brief Resilience Scale | Social Support Satisfaction | T1 CES Depression | T1 GAD Anxiety | T2 CES Depression | T2 GAD Anxiety |
| financial security | Pearson Correlation | 1 | -.097 | .004 | -.029 | **-.274^**^** | **-.155^*^** | -.084 | .097 | **-.244^**^** | **-.185^**^** | **.210^**^** | **.339^**^** | **-.278^**^** | **-.199^**^** | **-.321^**^** | **-.275^**^** |
|  | Sig. (2-tailed) |  | .170 | .960 | .685 | .000 | .028 | .233 | .169 | .000 | .008 | .003 | .000 | .000 | .005 | .000 | .000 |
|  | N | 202 | 202 | 202 | 195 | 202 | 200 | 202 | 202 | 202 | 202 | 200 | 201 | 200 | 200 | 168 | 168 |
| Age | Pearson Correlation | -.097 | 1 | **-.452^**^** | -.139 | -.080 | -.039 | **.496^**^** | **-.216^**^** | -.051 | **-.285^**^** | .009 | -.097 | -.021 | -.002 | -.125 | -.043 |
|  | Sig. (2-tailed) | .170 |  | .000 | .052 | .258 | .580 | .000 | .002 | .475 | .000 | .896 | .172 | .770 | .973 | .105 | .576 |
|  | N | 202 | 202 | 202 | 195 | 202 | 200 | 202 | 202 | 202 | 202 | 200 | 201 | 200 | 200 | 168 | 168 |
| Adolescent | Pearson Correlation | .004 | **-.452^**^** | 1 | .082 | -.043 | .086 | **-.304^**^** | -.068 | -.040 | .047 | -.026 | .020 | .032 | -.018 | **.161^*^** | .058 |
|  | Sig. (2-tailed) | .960 | .000 |  | .252 | .539 | .225 | .000 | .340 | .571 | .507 | .718 | .776 | .658 | .799 | .037 | .455 |
|  | N | 202 | 202 | 202 | 195 | 202 | 200 | 202 | 202 | 202 | 202 | 200 | 201 | 200 | 200 | 168 | 168 |
| Single Parent Status | Pearson Correlation | -.029 | -.139 | .082 | 1 | .122 | -.021 | **-.249^**^** | **.162^*^** | **.212^**^** | **.261^**^** | -.047 | -.115 | .135 | .100 | .066 | .072 |
|  | Sig. (2-tailed) | .685 | .052 | .252 |  | .088 | .775 | .000 | .024 | .003 | .000 | .512 | .111 | .061 | .167 | .402 | .364 |
|  | N | 195 | 195 | 195 | 195 | 195 | 193 | 195 | 195 | 195 | 195 | 193 | 194 | 193 | 193 | 162 | 162 |
| Negative Life Events: number | Pearson Correlation | **-.274^**^** | -.080 | -.043 | .122 | 1 | **.345^**^** | -.001 | .034 | **.141^*^** | **.194^**^** | **-.177^*^** | **-.219^**^** | **.413^**^** | **.393^**^** | **.350^**^** | **.348^**^** |
|  | Sig. (2-tailed) | .000 | .258 | .539 | .088 |  | .000 | .991 | .627 | .045 | .006 | .012 | .002 | .000 | .000 | .000 | .000 |
|  | N | 202 | 202 | 202 | 195 | 203 | 200 | 202 | 203 | 202 | 202 | 200 | 201 | 200 | 200 | 168 | 168 |
| ACES total | Pearson Correlation | **-.155^*^** | -.039 | .086 | -.021 | **.345^**^** | 1 | -.053 | .008 | .037 | .075 | **-.203^**^** | **-.309^**^** | **.341^**^** | **.359^**^** | **.265^**^** | **.294^**^** |
|  | Sig. (2-tailed) | .028 | .580 | .225 | .775 | .000 |  | .457 | .906 | .602 | .292 | .004 | .000 | .000 | .000 | .001 | .000 |
|  | N | 200 | 200 | 200 | 193 | 200 | 200 | 200 | 200 | 200 | 200 | 200 | 200 | 200 | 200 | 167 | 167 |
| Highest level of education completed: | Pearson Correlation | -.084 | **.496^**^** | **-.304^**^** | **-.249^**^** | -.001 | -.053 | 1 | -.118 | -.127 | **-.332^**^** | .002 | -.028 | -.065 | -.099 | -.093 | -.042 |
|  | Sig. (2-tailed) | .233 | .000 | .000 | .000 | .991 | .457 |  | .093 | .071 | .000 | .976 | .693 | .358 | .161 | .231 | .593 |
|  | N | 202 | 202 | 202 | 195 | 202 | 200 | 202 | 202 | 202 | 202 | 200 | 201 | 200 | 200 | 168 | 168 |
| Number of people in household | Pearson Correlation | .097 | **-.216^**^** | -.068 | **.162^*^** | .034 | .008 | -.118 | 1 | **.243^**^** | **.302^**^** | -.115 | -.038 | .109 | .017 | .108 | -.004 |
|  | Sig. (2-tailed) | .169 | .002 | .340 | .024 | .627 | .906 | .093 |  | .000 | .000 | .104 | .597 | .125 | .816 | .164 | .963 |
|  | N | 202 | 202 | 202 | 195 | 203 | 200 | 202 | 203 | 202 | 202 | 200 | 201 | 200 | 200 | 168 | 168 |
| Household Density (ppl/rooms) proportion score | Pearson Correlation | **-.244^**^** | -.051 | -.040 | **.212^**^** | **.141^*^** | .037 | -.127 | .243^**^ | 1 | **.207^**^** | -.026 | **-.212^**^** | .129 | .093 | .069 | .027 |
|  | Sig. (2-tailed) | .000 | .475 | .571 | .003 | .045 | .602 | .071 | .000 |  | .003 | .716 | .003 | .069 | .188 | .375 | .731 |
|  | N | 202 | 202 | 202 | 195 | 202 | 200 | 202 | 202 | 202 | 202 | 200 | 201 | 200 | 200 | 168 | 168 |
| Unstable Housing Status | Pearson Correlation | **-.185^**^** | **-.285^**^** | .047 | **.261^**^** | **.194^**^** | .075 | -.332^**^ | .302^**^ | .207^**^ | 1 | -.081 | -.053 | **.155^*^** | **.164^*^** | **.152^*^** | .144 |
|  | Sig. (2-tailed) | .008 | .000 | .507 | .000 | .006 | .292 | .000 | .000 | .003 |  | .253 | .456 | .028 | .020 | .050 | .063 |
|  | N | 202 | 202 | 202 | 195 | 202 | 200 | 202 | 202 | 202 | 202 | 200 | 201 | 200 | 200 | 168 | 168 |
| Brief Resilience Scale | Pearson Correlation | **.210^**^** | .009 | -.026 | -.047 | **-.177^*^** | -.203^**^ | .002 | -.115 | -.026 | -.081 | 1 | **.259^**^** | **-.529^**^** | **-.434^**^** | **-.347^**^** | **-.283^**^** |
|  | Sig. (2-tailed) | .003 | .896 | .718 | .512 | .012 | .004 | .976 | .104 | .716 | .253 |  | .000 | .000 | .000 | .000 | .000 |
|  | N | 200 | 200 | 200 | 193 | 200 | 200 | 200 | 200 | 200 | 200 | 200 | 200 | 200 | 200 | 167 | 167 |
| Social Support Satisfaction | Pearson Correlation | **.339^**^** | -.097 | .020 | -.115 | **-.219^**^** | -.309^**^ | -.028 | -.038 | -.212^**^ | -.053 | .259^**^ | 1 | **-.477^**^** | **-.383^**^** | **-.418^**^** | **-.356^**^** |
|  | Sig. (2-tailed) | .000 | .172 | .776 | .111 | .002 | .000 | .693 | .597 | .003 | .456 | .000 |  | .000 | .000 | .000 | .000 |
|  | N | 201 | 201 | 201 | 194 | 201 | 200 | 201 | 201 | 201 | 201 | 200 | 201 | 200 | 200 | 168 | 168 |
| T1 CES Depression | Pearson Correlation | **-.278^**^** | -.021 | .032 | .135 | **.413^**^** | .341^**^ | -.065 | .109 | .129 | .155^*^ | -.529^**^ | -.477^**^ | 1 | **.735^**^** | **.569^**^** | **.522^**^** |
|  | Sig. (2-tailed) | .000 | .770 | .658 | .061 | .000 | .000 | .358 | .125 | .069 | .028 | .000 | .000 |  | .000 | .000 | .000 |
|  | N | 200 | 200 | 200 | 193 | 200 | 200 | 200 | 200 | 200 | 200 | 200 | 200 | 200 | 200 | 167 | 167 |
| T1 GAD Anxiety | Pearson Correlation | **-.199^**^** | -.002 | -.018 | .100 | **.393^**^** | .359^**^ | -.099 | .017 | .093 | .164^*^ | -.434^**^ | -.383^**^ | .735^**^ | 1 | **.390^**^** | **.489^**^** |
|  | Sig. (2-tailed) | .005 | .973 | .799 | .167 | .000 | .000 | .161 | .816 | .188 | .020 | .000 | .000 | .000 |  | .000 | .000 |
|  | N | 200 | 200 | 200 | 193 | 200 | 200 | 200 | 200 | 200 | 200 | 200 | 200 | 200 | 200 | 167 | 167 |
| T2 CES Depression | Pearson Correlation | **-.321^**^** | -.125 | **.161^*^** | .066 | **.350^**^** | .265^**^ | -.093 | .108 | .069 | .152^*^ | -.347^**^ | -.418^**^ | .569^**^ | .390^**^ | 1 | **.782^**^** |
|  | Sig. (2-tailed) | .000 | .105 | .037 | .402 | .000 | .001 | .231 | .164 | .375 | .050 | .000 | .000 | .000 | .000 |  | .000 |
|  | N | 168 | 168 | 168 | 162 | 168 | 167 | 168 | 168 | 168 | 168 | 167 | 168 | 167 | 167 | 168 | 168 |
| T2 GAD Anxiety | Pearson Correlation | **-.275^**^** | -.043 | .058 | .072 | **.348^**^** | .294^**^ | -.042 | -.004 | .027 | .144 | -.283^**^ | -.356^**^ | .522^**^ | .489^**^ | .782^**^ | 1 |
|  | Sig. (2-tailed) | .000 | .576 | .455 | .364 | .000 | .000 | .593 | .963 | .731 | .063 | .000 | .000 | .000 | .000 | .000 |  |
|  | N | 168 | 168 | 168 | 162 | 168 | 167 | 168 | 168 | 168 | 168 | 167 | 168 | 167 | 167 | 168 | 168 |
| **. Correlation is significant at the 0.01 level (2-tailed).  *. Correlation is significant at the 0.05 level (2-tailed). | | | | | | | | | | | | | | | | | |
